# Bibliographical Notices

**Published:** 1838-01-31

**Authors:** 


### BIBLIOGRAPHICAL NOTICES.

*Mémoires de la Société Medicale d'observation, de Paris. Tome Premier.*

*Mémoire Analytique sur l'Orchite Blennorrhagique, par M. Marc d'Espine (de Génève) Docteur en Medicine, &c.*

*Memoirs of the Medical Society of Observation of Paris, Vol. 1. An Analytical Essay upon Blennorhagic Orchitis, by Dr. Marc D'Espine, of Geneva.*

(Concluded from page 27.)

The second part of M. D'Espine's memoir is occupied with a consideration of the causes and nature of blennorhagic orchitis. He treats first of the *remote* or *predisposing causes*. The number of circumstances susceptible of influencing mediately any affection are so numerous and so obscure, that a large collection of facts are necessary before we can safely hazard any induction. The propositions which our author advances are to be considered then more in the light of provisional results, whose intrinsic value is to be more accurately determined by future investigations, than as recognised laws.

*Age.*—In 29 cases 24 were under 30 years of age.

*Occupations.*—Those engaged in sedentary pursuits appear to be more liable to it than others whose habits of life are more active.

*Number of previous urethrites.*—14 out of 29 had had no previous attacks of gonorrhœa; the remaining 15 had suffered from one to four anterior attacks.

*Stage of the discharge at which orchitis most commonly occurs.*—Hunter, Swediaur and Faber affirm that it rarely or never happens at the commencement of the discharge. Bell on the contrary states that he has seen it at all periods of gonorrhœa indifferently. The result of our author's observations in 28 cases is as follows: During the 1st week of urethritis it happened in 2 persons; do. 2nd. 6; from the 15th to the 30th day, 6; from the 30th to the 60th day, 6; after the 60th day, 9.

To these we may add M. Guassail's table. During the 1st week, 3; 2nd week, 4; from the 15th to the 30th day, 21; 30th to 60th day, 39; after the 60th day, 6.

The other predisposing causes are ranged under the heads of temperaments, strength of constitution, anterior health, and previous syphilitic affections.

We next come to an examination of the *determining causes.* 25 individuals were examined with a view of ascertaining these. The result is as follows:

| | |
|---|---|
| Excess of muscular fatigue, - - | 12 |
| Ditto in wine and other alcoholic liquors, - | 3 |
| Use of remedies calculated to arrest the discharge, | 5 |
| Painful coition practised during an attack, - | 1 |
| Blows upon the testicles, - - - | 1 |
| Moral emotion (laughter,) - - - | 1 |
| Foreign body in the urethra, - - - | 1 |
| Laborious occupation, - - - - - | 1 |
| No appreciable cause, - - - - - | 4 |

Causes of relapses in 10 cases.

| | |
|---|---|
| Moderate exertion, but commenced too soon, - | 6 |
| Blow on the testes, - - - | 1 |
| Astringent injections, - - - | 1 |
| Unknown, - - - - - | 2 |

"The agreement of the results of this table with the preceding one is interesting. The same causes are present in both cases; the absence of some of them in the relapses is explained by the seclusion of the hospitals restraining the patients from excesses in wine, coition, the influence of professions," &c. p. 455.

The proportion of cases produced by muscular fatigue is 12-25; that of relapses from the same cause, is 6-10; the two results differ but slightly. In one-sixth of the cases orchitis supervened without any appreciable cause, and one-eighth of the relapses cannot be better explained. These results prove the great analogy between the original causes, and those of the relapses.

In a note M. D'Espine suggests the possibility of the practice of masturbation exercising an influence in the production of the primary attack as well as the relapses. He thinks it very likely that from the patients being generally individuals of singularly dissolute habits, on being deprived of their accustomed pleasures in the hospitals, they may resort to this disgusting vice. The secrecy with which it is practised, if the supposition be true, prevents our arriving at any definite conclusion. Would it not be worth while to investigate this cause more fully?

With respect to the presence of a foriegn body in the urethra as an excitant of orchitis, we believe it to be a very common cause, notwithstanding the solitary instance recorded by our author. Within a short time we have known it to happen twice from the introduction of a bougie in the treatment of stricture; and believe that the hospital experience of every surgeon will afford numerous analogous examples. M. D'Espine, we think, contradicts in the following sentence his own tabular statement, whilst at the same time he confirms our own opinion.

"If we consult not only our 23 cases of orchitis, but also 8 cases of anterior attacks, we find in both above 37 who recognise this as a cause."

*Of the suppression of the discharge considered as a cause of orchitis.*

"It is very important to determine the precise influence of the discharge in the development of orchitis, not only to illustrate the history of the disease, but also to throw light upon an essential question of general pathology, viz. whether metastasis may take place during the inflammatory period of any malady. Careful researches have led us strongly to doubt the existence of inflammatory metastasis. Clinical labors have enabled the better observers of our day to show that rheumatism, urticaria, erythema, affections in which metastasis plays so important a part, differ in so many points of view from maladies styled inflammatory, in which the same phenomena does not occur, that they are entitled to a separate classification. These works repose upon too solid a basis, to be invalidated by the purely systematic attacks of some physicians, who cannot submit to the entire guidance of facts." pp. 457.

We regret that our limits will not permit us to give more in detail M. D'Espine's investigation of this highly interesting and important part of our subject. The results however will be found in the subjoined extracts:

"To say that the commencement of blennorhagic orchitis is accompanied by a suppression or a diminution of the discharge, followed by a return or augmentation from the time that an amelioration of the symptoms occur, is to express that which happens at most three times out of 29, or in less than one-ninth of the cases, and is taking an exception for a rule.

"If on the contrary, this rule were true, it establishes a close connection between the two affections, and a dependence of one on the other, and would be a general fact worthy of notice. Ought we then to conclude that this relation although rare is real, and is something besides a mere coincidence? This is a delicate question and one I cannot finally determine; but it seems to me that sound philosophy ought to regard an *arrangement*, the nature of which expresses an intimate and peculiar result between two affections not oftener than once out of 9 or 10 times, as a mere coincidence, and not as cause and effect, or at least that judgment should be suspended upon it, and that we ought to be guided by presumptive evidence without deciding positively." p. 460.

From a careful examination of the facts presented by our author, as well as others, and after cautious consideration, on our part, of this subject, we feel much inclined to dispute the influence of the scare-crow metastasis in this as well as other affections, where so prominent a place is claimed for it in the etiological ranks. The diminution, or even suppression, of the urethral discharge we are disposed to consider as accidental, or coincident, or as perhaps indicative of an increase of the phlogosis in the canal. This latter supposition will not perhaps be considered so hypothetical when we recall *Bell's* idea of the origin of orchitis. He supposed it caused by an immediate extension of the inflammation along the urethra, vasa deferentia, into the body of the testis itself. M. D'Espine remarks:

"All the history of the *debut* of orchitis which I have given, supports strongly this opinion. *M. Guassail* is of the same mind, and his opinion is here the best authority that we can cite, since it is founded upon three autopsies of patients, dying of accidents and other diseases whilst suffering under orchitis."

In the late work of Lucas Champonniere, embodying the views of Cullerier, Surgeon of *Le Midi*, we find the same etiology advocated. The experience of Cullerier has been ample, and his reasoning, though perhaps not based on positive observation, is ingenious, and most likely agreeable to fact. The point however may be considered as still *sub-judice*, and one to which the Scotch verdict of *not-proven* would be probably returned by the strict disciples of the *methode numerique*. For ourselves we must confess a strong predilection in its favour, and a suspicion amounting almost to a conviction, that it is correct. Does not this view of the subject demand at least the careful attention of those surgeons, who commence their treatment of orchitis by the introduction of a bougie into the urethra, with the object of reinstating the discharge.

*The Treatment.*—We have now arrived at a division of the subject, which the majority of our readers will doubtless consider the most important. Let it be borne in mind however that our best directed therapeutic efforts repeatedly prove nugatory and vain without correct etiological and pathological views; that they all exercise a mutually reciprocal influence upon each other, and that a right understanding of the one is necessary to ensure the success of the other.

The *third* part of M. D'Espine's Memoir is occupied with the consideration of the treatment. At the onset we are advised that the therapeutic results are yet crude and unsatisfactory. The difficulties of arriving at precise results are rendered more perplexing from the fact, that the periods when the patients enter the hospital vary very materially; some resort to them early in the attack (*de boune heure,*) others again not until a much later time, having up to that moment pursued their wonted irregularities of life.

"The value of the systems of treatment," says our author, "is ever *relative*, and the standard of comparison should be a purely expectant plan, limited to securing repose, imposing diet, and at furthest administering some emollient tisans; we cannot hope to arrive at the complete solution of any therapeutic question, where the mode of treatment has been nearly uniform, and the number of *expectant* cases too few to enable us to make any positive judgment. However as the number of cases where local depletion was employed is sufficiently great, and as the use of this mean has varied on account of the number of leeches in one application, the comparison of these various modes will permit us to judge to a certain extent of their utility. Some patients having undergone, conjointly with the antiphlogistic treatment, that with inunctions of mercurial ointment, we shall discover whether this second treatment, added to the first, exercises a happy influence." pp. 468–9.

*Influence of early or late entrance into the Hospital.*—Tardy application for relief was found to prolong the duration of the affection, that of the pain, and the acknowledged period of the symptoms. The result of calculations made by D'Espine have convinced him that it is the three orders of symptoms just enumerated that are prejudiced by the cause we are studying.

| | | | | |
|---|---|---|---|---|
| One day of delay, | prolonged | duration of the | disease | 1-9 |
| do | do | do | pain | 1-28 |
| do | do | do | acute symptoms | 1-5 |

The most marked influence is exerted upon the duration of the acute period and the least upon that of the pain.

*Influence of Local Depletion.*—The duration of the disease appears to be shortened by an early use of leeches, and where a large number were employed in the first application. The leeches in every case were applied half upon the scrotum, and the remainder in the groin. If the pathological views we have advocated be correct, will not the application of leeches upon the scrotum, independent of other objections, tend to invite a flow of blood into the testicle; and would it not be better to limit their application to the course of the cord, and in the groin? In several instances we have seen the symptoms of orchitis, mild at the onset, become exasperated by the application of leeches upon the scrotum. In support of our own experience we may cite that of one of the most distinguished surgeons of this city, who has repeatedly witnessed the same thing.

*Of general blood-letting.*—This remedy was employed in three cases only, and the peculiar circumstances attending these will not permit us to arrive at any result.

*Influence of the application of Mercurial Ointment upon the scrotum conjoined to antiphlogistic treatment.*—No happy result was found to attend the adoption of this method; it was tried in four cases, and the duration of each exceeded the mean of all the rest. Salivation followed their use in one instance; the ointment had been rubbed on the scrotum for ten consecutive days.

*The hydriodate of potassa*, and the *protoiodide of mercury* were used in several cases. Frictions on the scrotum with the former in two instances appeared to exercise some influence in hastening resolution.

Cataplasms of flaxseed meal with, or without, the addition of laudanum, were used in every instance. They were ordinarily limited to the acute stage, and were then, in the majority of cases replaced by an oleo-camphorated liniment. The poultices seemed to exert a favorable effect upon the scrotum in the first days of their application, especially when often renewed. Their action and particularly a prolonged use of them, produced sometimes a redness of the scrotum, different from that caused by the disease, more violet, and resembling the natural colour of the part. Sometimes a papular eruption, accompanied with intense itching, was the result of these topics. The œdematous condition of the integuments of the scrotum without heat, previously mentioned, appears sometimes to be caused by a continued use of poultices, but more especially by that of lead water. This in one case was particularly conspicuous.

The camphor liniment was resorted to with a view of preventing erections, and any new excitation of the genital organs, and thus to avoid relapses. Whether these anticipations were realised it is impossible with any certainty to determine.

With respect to the influence of the treatment of gonorrhœa during the existence of orchitis, upon the malady, M. D'Espine thinks that we are justified in attempting to arrest the discharge before the complete disappearance of orchitis. To effect this he advises the employment of internal medications, as copaiba, cubebs, *&c.* instead of astringent injections. This opinion is derived from the results of 8 cases, 7 of which were treated by the balsams, and 1 by injections. The latter case was followed by a relapse, supposed to be in consequence of the astringent used.

We have now presented our readers with a concise outline of M. D'Espine's Memoir. Every one who peruses the original must be convinced of the unwearied industry, rigid analysis and cautious induction which characterises the labours of our author. There is a total absence indeed of that offensive verbosity, and tedious and unprofitable detail which disfigure the majority of French works. From the number of observations in many instances being limited, the results are occasionally far from satisfactory. As the results are founded on the broad and stable basis of facts, and not on the fanciful speculations of theory, this could not be avoided, and is an instance of the scrupulous fidelity with which M. D'Espine has discharged his task. The conclusions as far as they go, are of immense value, and may be viewed as established laws. Further investigations conducted with the same commendable and persevering spirit will conduce much towards perfecting our knowledge of this interesting affection. To the labours and services of the School, of which M. D'Espine is a member, in the advancement of the holy cause of truth, too much praise and gratitude cannot be awarded. Since their work has commenced a new era has dawned upon medical science. Systems once deemed inviolate, whose errors were unknown, because hidden by the accumulated dust of centuries, have fallen before the steady onward march of *facts;* and opinions looked upon as suspicious and apochryphal, have by the same means, been recognised as axioms.

For ourselves individually, though prepared to acknowledge fully the immense advantages and superiority of the *numerical method* in all the departments of medicine, we are inclined somewhat to doubt that exclusive applicability of it to therapeutics, so warmly claimed for it by its advocates. As we have no two individuals alike, so are no two diseases precisely similar throughout their whole course. To reduce therapeutics to any thing approaching to certainty, much time, and many more observations are demanded. For awhile yet our practice in many respects must be empirical though we should never loose sight of *principles.* Guided by these and the pure light of the Baconian Philosophy—*numerandæ et perpendendæ observationes*—we shall every day arrive nearer the goal of perfection.

---

*The Philadelphia Practice of Midwifery, by* Charles D. Meigs, M. D., *Lecturer on Midwifery, &c. &c.—with numerous engravings. Philadelphia: James Kay, Jun. and Brother,* 1838. 12*mo. pp.* 370.

This work is an elementary treatise on midwifery, based upon a long clinical experience of a most successful practitioner. Within the pages of a duodecimo volume is compressed a lucid explanation of the principles and routine practice of the obstetric art, with a copious citation of interesting cases, from the note book of the author. The mechanism of labour is treated with a fulness, that contrasts favourably with the meagre details upon this subject, in a late British publication, (Collins' Midwifery.) The clinical illustrations, which Dr. Meigs has interspersed through his chapters, form an interesting portion of the work. They are selected with great happiness, and will be received as a most valuable contribution to the science of obstetrics. We may particularize the history of a case of labour in a deformed pelvis, which Dr. Meigs designates, justly we think, as "probably the most difficult obstetric operation, ever performed in this country." The details of this case, (the lady who underwent the operation of hysterotomy, noticed in our first number,) were drawn up by Dr. George Fox and published originally in the North American Medical and Surgical Journal.

The chapter on *atresia vaginæ* contains the record of a most remarkable instance of this affection, under the care of Dr. Meigs, in conjunction with Dr. Randolph. The case was one of very rapid delivery, followed by severe inflammation and sloughing of the vagina, terminating in complete occlusion. An operation was attempted for the relief of the distress consequent upon the accumulation of the menstrual fluid, which proved eminently successful. The lady now menstruates *per vias naturales,* and is entirely restored to health. A case of atresia occurred in the Pennsylvania Hospital about eighteen months since, in a black woman, 28 years of age, on whom Dr. Randolph operated, but without the same beneficial results that attended the case, mentioned by Dr. Meigs.

The style of the "Practice of Midwifery," is lively and attractive. Apart from its intrinsic merit, it is most agreeable reading, a very pleasant set off against the heavy, matter of fact strain, that pervades too many medical works of the day. The student and practitioner of midwifery will find it a most instructive and entertaining manual.

---

*An oration on the Improvements in Medicine, delivered before the Philadelphia Medical Society, twelfth month,* 13*th,* 1837. *By Joseph Warrington, M. D. Honorary Member of the Society, and Accoucheur to the Philadelphia Dispensary. Published by order of the Society,* 8*vo. pp.* 28. *Philadelphia,* 1837.

Dr. Warrington has selected a subject as ample and varied, as it is hackneyed and trite. He has however done full justice to it. The language is chaste and appropriate; and the style is in general free from rhetorical effort, the besetting sin of the medical orations of the period.

